# Were the First Trace Fossils Really Burrows or Could They Have Been Made by Sediment-Displacive Chemosymbiotic Organisms?

**DOI:** 10.3390/life12020136

**Published:** 2022-01-18

**Authors:** Duncan McIlroy

**Affiliations:** Earth Sciences & Bonne Bay Marine Station, Memorial University of Newfoundland, St. John’s, NL A0K 3V0, Canada; dmcilroy@mun.ca

**Keywords:** graphoglyptid, rangeomorph, Ediacaran, Cambrian, trace fossil, ichnology, Chemosymbiont, burrowing, palaeobiology

## Abstract

This review asks some hard questions about what the enigmatic graphoglyptid trace fossils are, documents some of their early fossil record from the Ediacaran–Cambrian transition and explores the idea that they may not have been fossils at all. Most researchers have considered the Graphoglyptida to have had a microbial-farming mode of life similar to that proposed for the fractal Ediacaran Rangeomorpha. This begs the question “What are the Graphoglyptida if not the Rangeomorpha persevering” and if so then “What if…?”. This provocative idea has at its roots some fundamental questions about how to distinguish burrows sensu-stricto from the external molds of endobenthic sediment displacive organisms.

## 1. Introduction

The importance of the first colonization of the sedimentary realm by infaunal organisms has been at the heart of discussions around the evolution of complex animal life and the beginning of the Cambrian Explosion of animal life [1,2,3,4]. The base of the Cambrian period (and end of the Ediacaran) is defined by the first occurrence of trace fossils belonging to the *Treptichnus pedum* Assemblage Zone [5,6] at a point in rock in Fortune Head in Newfoundland, Canada approx. 540 Ma. The precept behind this decision was the recognition that burrowing is an easily preservable—fundamentally animalian—trait either in the form of fossil burrows or burrowing fabrics [1,7].

It has become increasingly clear in recent years that complex animals evolved well before the base of the Cambrian. Indeed, recent studies consider two of the major Ediacaran clades (the Arboreomorpha and Rangeomorpha) as members of total group Eumetazoa [8]. Evidence for the existence of Ediacaran animals includes: preserved cnidarian muscles (in the staurozoan-like *Haootia* [9,10]) and surface locomotion trails [11] both from around 565 Ma; the mollusk-like grazing trace *Kimberichnus* [12] c. 550 Ma; serial impressions of placozoan-type feeding (*Dickinsonia, Epibaion* [13,14,15] c. 550 Ma; as well as bilaterian burrows [16] and possible annelid trails [17] close to the basal Cambrian both c. 542 Ma. Debates around whether the Cambrian explosion of complex animal life had a short or long Ediacaran fuse [18,19] have thus mostly converged on a consensus that there was a long Ediacaran pre-history to the Cambrian biotas. The issue of how and why complex animal life diverged so markedly during the Ediacaran–Cambrian transition is still a source of debate [20,21,22,23,24,25,26,27,28].

Perhaps the most interesting questions around Ediacaran paleobiology relate to first and last occurrences of taxa [9,29,30,31], but also the biotic transition from the Ediacaran into the Cambrian [26]. With almost all first order evolutionary innovations (e.g., biomineralization, terrestrialization, etc.) there is a period of time with equivocal evidence for the event prior to its universally accepted advent. This is likely usually due to localized innovation that is difficult to characterize, followed by rapid radiation/dispersal [32]. The record of the end of the Ediacaran and the Cambrian explosion of animal life includes examples of Ediacaran survivors in Cambrian rocks (e.g., *Swarpuntia* [33,34]), and also evidence of putative Cambrian type trace fossils below the recognized Ediacaran Cambrian boundary [35,36,37]. It is to this latter transition, from the matground dominated Ediacaran to the macroscopically bioturbated Cambrian [2,38] that our attention is drawn herein.

## 2. Microbially Dominated Seafloors at the Dawn of Animal Life

**Figure 1:**
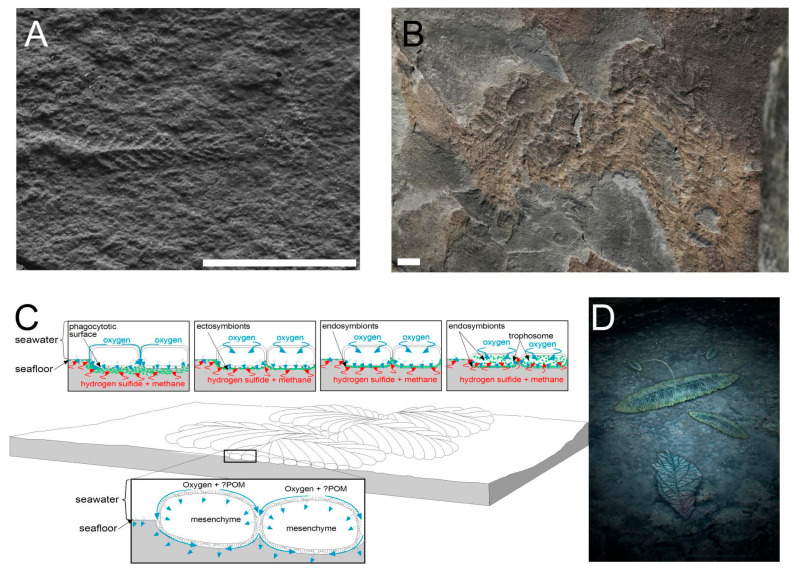
(**A**) Long, narrow Ediacaran frond from Mistaken Point Ecological reserve, NL (scale bar in mm); (**B**) large reclining rangeomorph Ediacaran frond. aff. *Bradgatia* sp. from the MUN surface, Catalina Dome, NL; (**C**) diagrammatic reconstruction of a generic reclining rangeomorph detailing the ways that it might have interacted with the substrate. The lower surface is irrigated with seawater by ciliary action and diffusion. The supply of oxygen to the lower surface is considered to have increased microbial productivity. The top row of images shows possible feeding modes with green circles showing the distribution of chemolithoautotrophic symbionts and arrows show diffusion of solutes. Furthest left is phagotrophy, next is ectosymbiosis, then endosymbiosis and furthest right is endosymbiosis with a trophosome (requiring diffusion of sulfide/methane into a thin organism and POM = particulate organic matter). All of these methods of gaining nutrition would work for endobenthic graphoglyptids; (**D**) reconstruction of the Ediacaran seafloor of Mistaken Point Formation (courtesy of Paleocreations).

Matgrounds were common in late Proterozoic marine ecosystems, forming wherever there was a sufficiently low rate of sedimentation to allow organic matter to settle onto sediment surfaces. In the absence of surficial detritus-feeders and conveyor activity by bioturbators, the development of matgrounds developed largely unchecked for the majority of the Proterozoic history of microbial life [39]. The microbial consortia that made up Proterozoic and lowermost Palaeozoic matgrounds, the physical integrity of matgrounds, and their shear strength remain effectively unknown. It is presumed that in shallow water depositional settings there was a strong photosynthetic component and that matgrounds were dominantly algal in nature [40], but in deep marine settings the matgrounds likely also had a range of sulphur-oxidizing bacteria close to the sediment-water interface [41,42]. Modern matgrounds are loci of large amounts of microbial biomass and microbial dissolved organic matter (DOM) production [43]. In fine-grained sediments, the matground microbiota occludes pore throats with filaments, resulting in porewater dysoxia or even anoxia very close to the sediment-water interface [44]. The fine-grained sediment below the (macro)fossiliferous Ediacaran matgrounds of Avalonia was most commonly pelagite or hemipelagite, probably with relatively high amounts of porewater [45,46]. The smothering of these matground surfaces by the growth of reclining organisms or fallen erect organisms commonly caused the preservation of negative impressions of even the delicate fronds of Ediacaran organisms [15] (Figure 1A).

One of the most distinctive aspects of the earliest Ediacaran soft-bodied macrobiotas is that—with few rare exceptions—they were immotile, and in many cases grew to very large sizes on matgrounds [29,47,48] (Figure 1B). Being immotile on a porous organic-rich seafloor potentially results in serious biogeochemical challenges in the form of hydrogen sulfide buildup below the body tissues [49,50,51]. If hydrogen sulfide accumulates unchecked next to the epithelium of an immotile recliner it would likely cause cell-death, meaning that soft-bodied Ediacaran organisms must have been able to modify the organism-substrate interface in a manner that detoxified, or otherwise mitigated, sulfide toxicity [52]. Other strategies that animals employ to allow growth on sulfidic porewater substrates involve creation of an inert barrier between the sediment and the organism such as the holdfasts of crinoids [53], the basipinacocytes of sponges [51], or the mucous burrow linings of burrowers that make permanent dwellings (e.g., cerianthid anemones [54,55]). The most common way for modern soft-bodied organisms to avoid sulfide toxicity is to either move on a regular basis (e.g., the placozoan *Trichoplax* initiates movement in response to sulfide concentrations [56]) or to detoxify this hydrogen sulfide by pumping oxygen to the sediment interface, causing oxidation of sulfide to thiosulphate [50,57] (Figure 1C). There are many common ecto- and endo-symbioses between sulfur oxidizing bacteria and animals, particularly on high surface area, oxygen-rich, epithelia such as gills [58,59].

The earliest examples of Ediacaran fossils include the epibenthic Rangeomorpha, some of which had fractal-like lower surfaces and lived reclined on the seafloor [52,60,61] (Figure 1D). Some rangeomorphs actively displaced sediment during growth such that they grew slightly below the ambient sediment–water interface [62] and as such were likely adapted to exploit sedimentary biogeochemical gradients, especially the very large reclining organisms (e.g., *Bradgatia* [47] and *Gigarimaneta* [48]). Fractal-like morphologies in reclining organisms are most consistent with sedimentary nutrient exploitation via symbioses with lithoautotrophic bacteria, based around the metabolism of methane, hydrogen, and hydrogen sulfide in particular. In these symbioses, the rangeomorph probably provided oxygen to and gained nutriment from the symbionts that it hosted. It is most likely in these simple organisms that there was a mixture of symbiosis and phagocytosis on the lower surface of the organism, in the microbial productivity hotspot generated by the localized enhanced near-organism oxic zone [50] (Figure 1C).

Due to the low rate of diffusion of oxygen into the sediment porewater systems that underlay the ubiquitous Ediacaran seafloor matgrounds, the redox profile of Ediacaran sediments is likely to have been significantly condensed [2,63,64,65,66]. As a result, very little of the particulate and dissolved organic matter in such sub-mat settings will have been subject to aerobic respiration (the greatest energy yield per unit of organic carbon metabolized [67,68]), leading to a predominance of sulfate reduction and methanogenesis. However, should a reclining organism grow atop an established matground and pump oxygenated seawater to its lower surface, this would stimulate productivity of chemolithoautotrophic bacteria such as sulfur oxidizers (which could utilize reductants diffusing from the sub-mat sediment profile, e.g., HS^−^, NH_4_^+^, Fe (II) [69]) as well as methanotrophs [70]. Such stimulation of microbial productivity is likely to have constituted the basis for simple ectosymbiosis/phagotrophic nutrition for reclining macro-organisms [61].

## 3. The Slow Death of the Ediacaran-Type Matground Biotope

From their acme in the Proterozoic, matgrounds such as stromatolites slowly declined, becoming increasingly marginalized in the lowermost Paleozoic [71]. Paleozoic matground facies became increasingly associated with environments that were somewhat hostile to burrowing animals such as low TOC mud-belts in front of deltas [46,72], whereas in the lowermost Cambrian matgrounds were common in normal marine settings such as the lower shoreface [2]. Evidence for matground facies in siliciclastic settings is commonly in the form of microbially induced sedimentary structures (MISS) such as lineated bedding planes of Arumberia, wrinkled surfaces such as Kinneya and elephant-skin textures [41,73,74,75,76,77]. These same textures commonly recur after mass extinction events until biotic recovery re-establishes ecosystem services in the benthic realm, including the all-important ecosystem engineering burrowing endobenthos [78,79,80,81,82].

**Figure 2:**
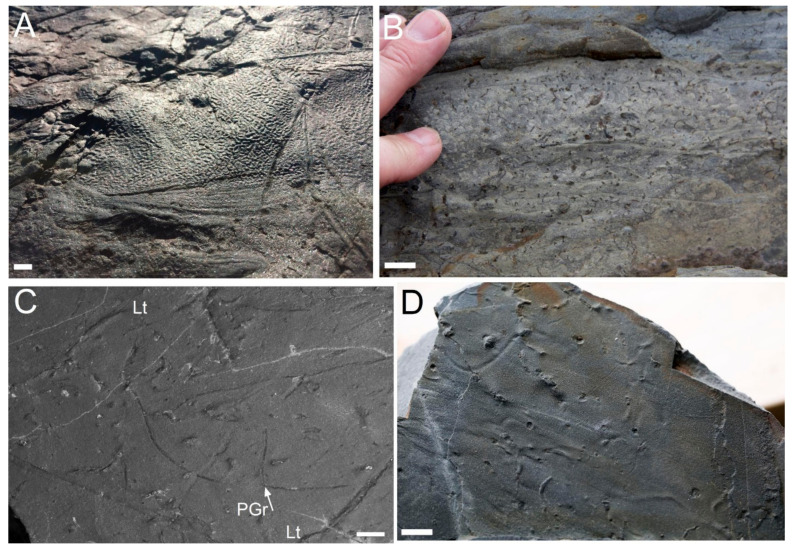
(**A**) Microbial matground surface with wrinkled texture and abundant sediment mining trace fossils from the Ordovician of Bell Island, NL; (**B**) typical ichnofabric from the lower Fortunian of Fortune Head showing abundant curved, spiraling and branching pyritized burrows; (**C**,**D**) bedding plane view of *Lamonte trevallis* burrows (Lt) and pyritized graphoglyptid burrows (PGr) with T junction arrowed. Scale bars 1 cm.

The stresses on the matground biotope that dominated hiatal marine seafloors of the Proterozoic largely result from the effects of bioturbation, which seemingly started in the Ediacaran with the evolution of bilaterian burrowers [16] along with the grazing activity of metazoans [12,17,83]. This matground stress likely escalated with the evolution of larger bulk-sediment deposit feeders around the base of Cambrian Stage 2 [80], becoming better established as bioturbators increasingly sought out surficial and buried organic rich substrates through the lower Palaeozoic (Figure 2A). Modern levels of bioturbation and distribution would likely have developed very quickly.

The presence of shallow burrows co-existing with elements of the soft bodied Ediacaran biota, while not entirely unexpected, does need to be considered with an open mind to alternative hypotheses. The morphologies of late Ediacaran burrows are commonly simple and narrow (Figure 2B). The most abundant trace in this period is the simple tubular burrow *Lamonte trevallis* [84] (Figure 2C,D)*,* which is interpreted as a member of an ichnoguild of under-mat miners [85]. Other regularly serial or branched burrows are commonly attributed to the treptichnid genera *Treptichnus* and *Streptichnus* [36,37,86]. The importance of identifying *Treptichnus* alongside elements of the Ediacaran biota stems from the fact that the *Treptichnus pedum* (originally *Phycodes pedum*) ichnoassemblage zone is diagnostic of the base of the Cambrian, thereby creating an apparent stratigraphic conundrum. Though in the present author’s opinion, none of the purported Ediacaran *Treptichnus* closely resembles *T. pedum*, typically being very thin with narrow angle of branching. This begs the question—to me at least—if they are not *Treptichnus* s.s. then what might they be?

## 4. The Early Putative Burrowers of the Ediacaran–Cambrian Transition

It is a seldom appreciated precept of ichnological (trace-fossil) studies that burrows do not generally betray the taxonomic affinities of the burrowing organism [87], nor do they always represent a single life activity in most cases [55]. A simple vertical burrow in a sand, for example, works just as well as a den for a predator or mucous net feeder as it does for a head-down deposit feeder [88]. While most biologists would accept that as a truism, many palaeo-ichnologists are surprisingly content with making broad-brush assumptions of behavior based on burrow morphology [89].

**Figure 3:**
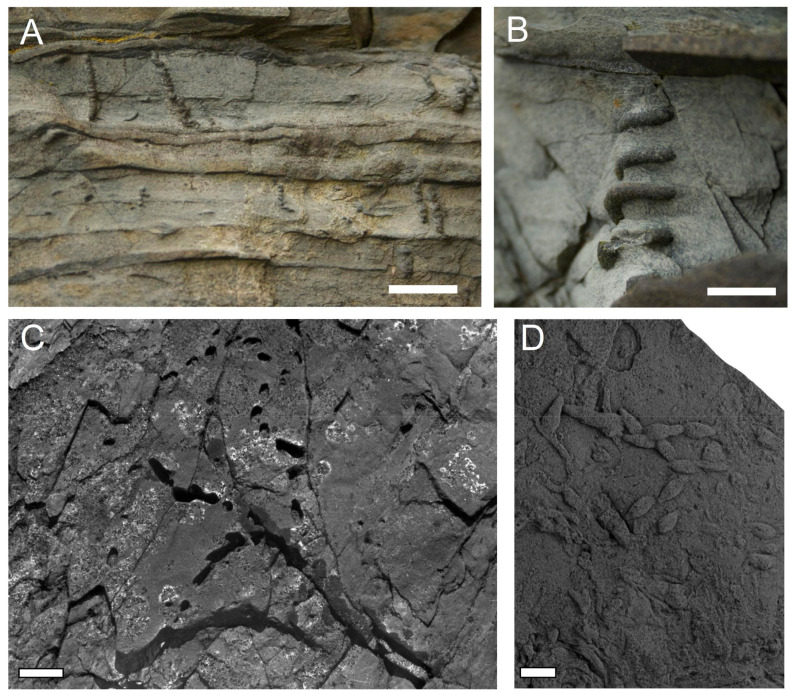
Tubular open “burrows” from the Fortunian of Fortune Head NL showing spiraling morphologies of: (**A**) *Gyrolithes gyratus*; and (**B**) *G. scintillus* with pyrite rich silty sandstone fill. (**C**) Shows the bedding plane view of a uniserially branching *Treptichnus pedum* in which the pyritic fill has weathered away showing the mold of the burrow, the space that would have been occupied in life. Whether these structures were burrows sensu-stricto or casts of the exterior of spiraling or branching organisms remains to be determined. (**D**) Natural sandstone cast of *T. pedum* from the Arumbera Formation in conventional positive hyporelief Scale bars 1 cm.

The majority of the earliest fossil burrows do not show good evidence for deposit feeding activity, but rather are passively sediment-filled, diagenetic mineral-filled, or collapsed [80]. In the type of section for the Ediacaran–Cambrian boundary in southeastern Newfoundland, Canada, the open, passive filled burrows *Treptichnus* and *Gyrolithes,* dominate the ichnology of the Fortunian-aged *Treptichnus pedum* assemblage ichnozone [1,2,80,90] (Figure 3A–D). In the Fortunian stage of the lower Cambrian there are also abundant surface traces including arthropod burrows and surficial grazers/bulldozers [1,91]. It is not until slightly higher in the lower Cambrian (Cambrian Stage 2) that there is unequivocal evidence of bulk sediment deposit feeding activity [2,3,80].

**Figure 4:**
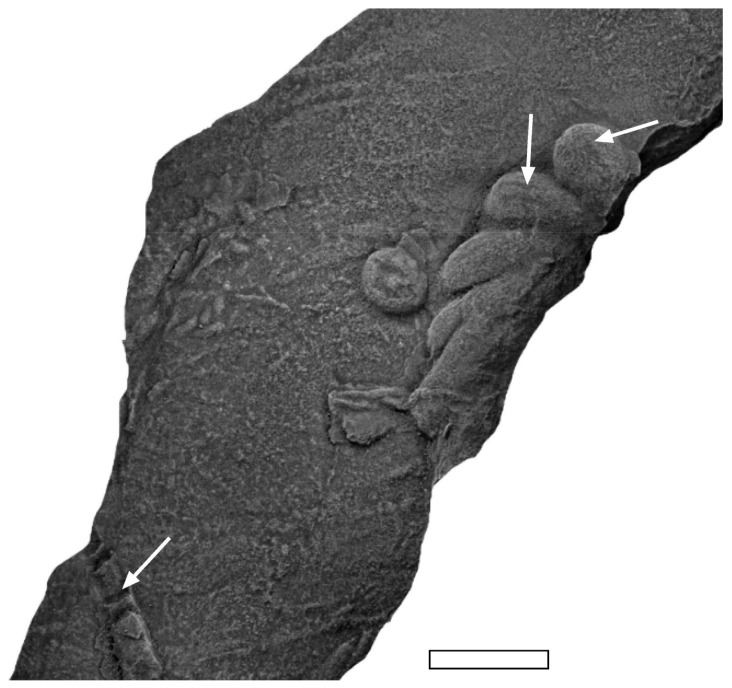
Segments of *Trichophycus* ispp. from the lower Cambrian Arumbera Sandstone of central Australia showing the stacked spreite (arrowed bottom left) and scratch marks (arrowed top right) that distinguish the genus from *Treptichnus*. Scale bar 1 cm.

The ichnogenus *Treptichnus* was created for fossilized burrows [92] and has subsequently been applied to a range of marine trace fossils from deep marine turbidite successions throughout the Phanerozoic, as well as shallow marine trace fossils of the Palaeozoic and burrows of modern insect larvae [93]. The generic diagnoses of the similarly branching burrows of *Trichophycus* and *Phycodes* include the formation of spreite by serial bulk sediment deposit feeding and direct evidence of movement in the form of bioglyphs; fecal pellets are known from Cambrian Stage 2 [94,95] (Figure 4). Both *Trichophycus* and *Phycodes* have *Treptichnus-*like biserial and uniserial branching, which is almost certainly an example of convergent behavioral evolution for effective sediment exploration and exploitation using sympodial/feather stitch branching [3,4,96].

While the ichnotaxonomic minutiae have been explored in detail, the question that seems not to have been asked is: what evidence do we have for the behavior represented by the lowermost Cambrian marine treptichnids? We know that organisms have been able to exploit sub-seafloor settings by sediment displacive growth since the Ediacaran [62], so the question remains “Do we even know if the earliest endogenic structures were trace fossils sensu-stricto and not just external molds of the first sediment displacive endobenthos?” I would posit that perhaps we do not.

If we are to open ourselves to the possibility of sediment displacive growth [62] persisting beyond the Ediacaran, then there are a wide range of lower Cambrian burrow-like structures that are always passively filled with sediment or collapse (i.e., not backfilled by the trace maker) that could be reinvestigated. In this case rather than being burrows we could think of them as external molds.

Note that this is not the same as the approach to *Treptichnus pedum* by Dzik [23] who conflated biotaxa and ichnotaxa (creating a priapulid genus *Manycodes*), even though the two do not complete under the ICZN. *Manycodes* has not been accepted as being synonymous with *Treptichnus*, though the Scalidophora are considered a likely trace-makers of *Treptichnus*- and *Trichophycus*-like burrows both modern and ancient [97].

## 5. What are the Graphoglyptida if not the Rangeomorpha Persevering?

One of the remarkable things about the “trace fossil” record of the shallow marine matground-rich facies of the lowermost Cambrian is that there are numerous narrow, geometric graphoglyptids [97,98]. Graphoglyptids are primarily known from deep marine depositional settings [99,100,101] (but sometimes shallow marine as well [102,103]) from the Ordovician onwards and having a major radiation in the Cretaceous [101], perhaps coincident with the expansion of deciduous trees and grasslands. The affinities of the Graphoglyptida are contentious, and even though some examples are known from modern seafloors, no trace-maker has yet been positively identified [104].

**Figure 5:**
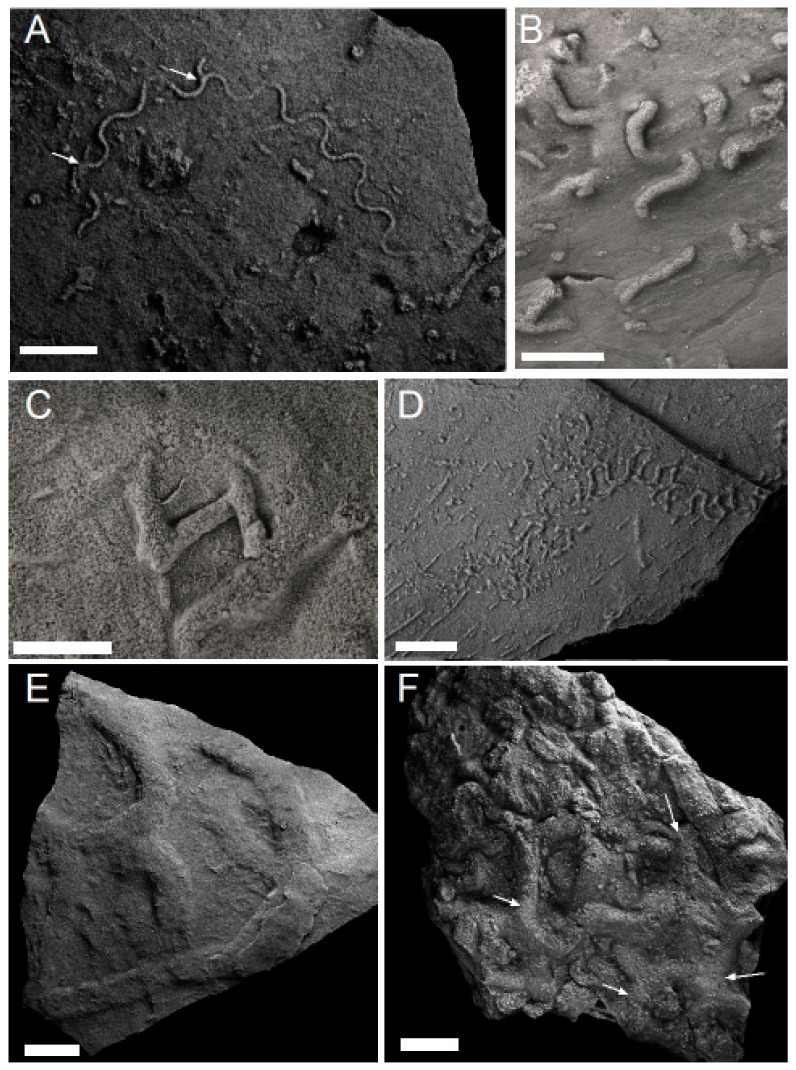
Graphoglyptid morphologies: (**A**,**B**) are line graphoglyptids ((**A**) *Helminthoida* though note the branching from the Cambrian Arumbera Sandstone Australia; (**B**) is *Helicolithus* from the latest Ediacaran of Tanafjord, Norway); (**C**,**D**) are branching graphoglytids. ((**C**) is *Belorhaphe* from the late Ediacaran of Tanafjord, Norway, (**D**) is cf. *Paleomeandron* from the Cambrian Arumbera Sandstone); (**E**,**F**) are net graphoglyptids ((**E**) is *Squamodictyon* and (**F**) is *Paleodictyon* from the Arumbera Sandstone, Australia). Scale bars 1 cm.

Recent work has divided the Graphoglyptida into three topological groups [105]: (1) “line graphoglyptids” (mostly meanders and spirals) which are common in the Fortunian lower Cambrian worldwide (Figure 5A,B); (2) “tree-form (mainly sympodially-branching) graphoglyptids” (including *Treptichnus* [106]) which are locally common in lower shoreface settings (Figure 5C,D); and (3) “net-type graphoglyptids” that are generally rare except in tempestite and prodelta turbidite deposits [1,3,107] (Figure 5E,F).

Most authors have considered the mode of life of the graphoglyptid-making organisms to include a combination of: (1) intensive [near]surficial bulk-sediment detritus feeding in meanders and spirals [98,105]; and (2) the creation of open sub-surface branching burrows and networks that were maintained for the purpose of “farming” microbes on the burrow wall [98].

### 5.1. The Early Vermiform/Line Graphoglyptids

In the Cambrian, hiatal matground facies prior to the onset of deep deposit feeding activity is likely to have been associated with surficial-concentrated nutrients similar to the distribution of food on the deep basin floors exploited by modern systematic (meandering/spiraling) deposit feeders [107]. The similar trace fossil assemblage is perhaps to be expected.

The surficial matground biotope was host to some of the earliest Ediacaran endogenic structures (e.g., *Lamonte trevallis*). The ability of the organisms to penetrate matground textures is a most surprising and fundamental innovation, potentially opening up the sub-matground porewater systems to a second phase of microbial oxidation of buried organic matter [108]. Since backfill is yet to be demonstrated in this under-mat-miner guild, it should also be considered that the open tubular structures with their high surface area to volume ratio might have been suitable for cilial bioirrigation by a very simple immotile animal living in the sediment. Such a mode of life would be particularly effective if the *Lamonte*-making organism had symbionts as did some of the rangeomorphs.

Other similar, open, unbranched features described as burrows are common in the latest Ediacaran and lower Cambrian. Several distinctive spiraled/sinuous taxa of uniform diameter without backfill are known from within meters of the Ediacaran–Cambrian boundary, including the vertically spiraled *Gyrolithes scintillus* and *G. gyratus* (Figure 3) and horizontally spiraled *Helicolithus* [3,80,90,109] (Figure 5A), *Streptichnus* [86], and some prossible *Treptichnus* [37]. All of these taxa are considered to have been maintained such that they were constantly open to seawater and are commonly partly pyritized. That the burrows are commonly pyritized is suggestive of the presence of sulfur oxidizing bacteria that would be predicted by the ciliary irrigating mode of life of the symbiotic/phagocytotic Rangeomorpha proposed by Dufour and McIlroy [50].

Previous work has noted the potential for bacterial farming in *Gyrolithes* [90], presumably via bioirrigation [88], but did not consider a rangeomorph-like chemosymbiotic-phagocytotic mode of life. The bacterial farming mode of life seems to rely on some form of burrow wall grazing for which there is to date no convincing evidence. Younger occurrences of *Gyrolithes* are commonly attributed to conventional dwelling or deposit feeding burrows of bilaterian taxa from various “worms”, arthropods and even vertebrates [90]. Modern *Helicolithus*-like burrows are known to be formed in sulfidic marine sediments by the deposit feeding enteropneust *Saccoglossus* [110].

If the paradigm for a rangeomorph-like symbiotic lifestyle can be extended to unbranched, high surface area-volume ratio burrows without evidence of burrowing action/feeding, then the atypical nature of the earliest trace-fossil biotas and their overlap with the Ediacaran biotas might be explainable.

### 5.2. The Tree-Like Graphoglyptids of the E-C Boundary

Recognition of the tree-like graphoglyptids in bedding plane expression is commonly facilitated by the presence of sharp, commonly high angle branches—even 90° branching. That in itself is unremarkable [111], but to have 90° branching without corner rounding (see *Treptichnus* in [87], their Figure 6) is unusual/unknown in burrows that are constantly patrolled by the trace-maker. A large number of trace fossils fall into this category; many of them are very beautiful, consisting of high angle branching in complex shapes, often forming meanders and almost never self-crossing. Most Phanerozoic examples of the tree-like graphoglyptids have very long chains of self-similar elements in a single meandering burrow [101]. Cambrian examples attributed to the same ichnotaxa tend to be short and slightly atypically irregular [3] (Figure 5C,D).

In the farming model for graphoglyptid paleobiology [98], the endobenthic organism is inferred to have either actively or passively irrigated the burrow, thereby providing a large surface area supplied with oxygenated seawater upon which a microbiota could be cultured.

**Figure 6:**
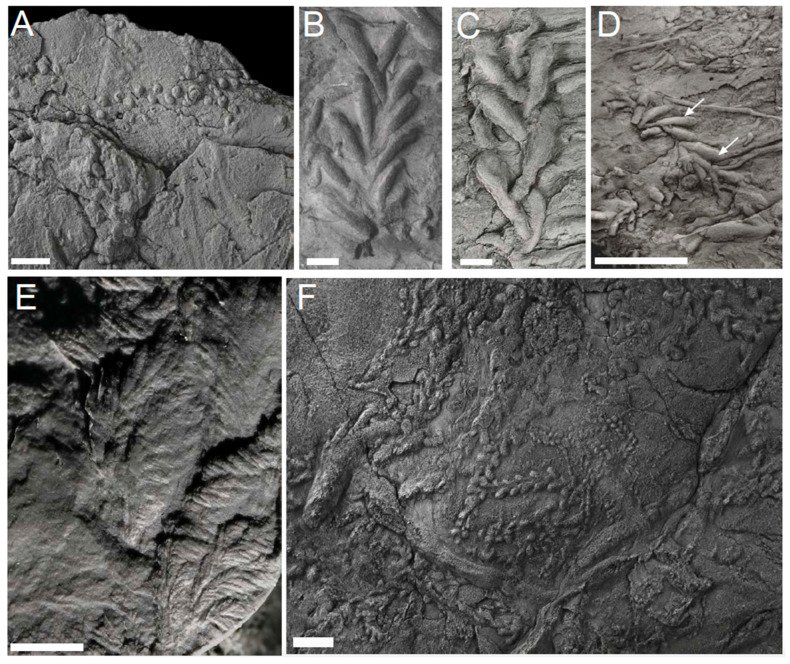
(**A**–**D**) *Treptichnus pedum* showing a range of branching types. All preserved as open burrow fills from the Fortunian of Tanafjord, Norway. D shows both uniserial and biserial monopodial branching. (**E**) is a small portion of aff. *Bradgatia* showing *Treptichnus* such as branching. (**F**) *Treptichnus lublinensis* showing meandering habit and very rangeomorph-like branching. Scale bars 1 cm except (**D**), which is 5 cm.

The most common tree-like, branching open burrow in the Cambrian is *Treptichnus pedum*, which may have alternated between biserial and uniserial sympodial branching. The feather-stitch biserial branching produces effectively straight burrows, with terminal openings at the end of each blind ended branch (Figure 6A). The length of branches and their angle can vary considerably, affecting spacing between branching (Figure 6B). The same burrows can curve by undergoing uniserial sympodial branching (Figure 6C) while avoiding self-crossing, perhaps in response of physico-chemical seafloor gradients.

The epibenthic rangeomorph *Bradgatia* undergoes similar branching in search of nutrients (Figure 6D) and likely had an oxygen-capturing upper surface and a ciliated lower surface providing fresh supplies of seawater to its episymbionts. The other species of *Treptichnus* that is only known from the lower Cambrian is the very shallow tier rangeomorph-like *Treptichnus lublinensis*, which would not look out of place in some of the iconic deep marine Ediacaran biotas [52] (Figure 6E). Additionally, zig-zagged open burrows attributed to *Belorhaphe* isp. (Figure 5C) from the latest Ediacaran of Norway [3] are similar to *Treptichus* except for the branching position and small size. This not to say that the treptichnids and forms such as *Belorhaphe* were indeed rangeomorphs per-se, just that they may have had a rather rangeomorph-like mode of life and growth (albeit endobenthically rather than epibenthically) and were not necessarily deposit feeders as is commonly stated but may have had a sediment-displacive mode of life. As we strive to understand these purported trace fossils, we need to bear in mind the possibility that they could be external molds rather than burrows.

### 5.3. The Net-Like Graphoglyptida

**Figure 7:**
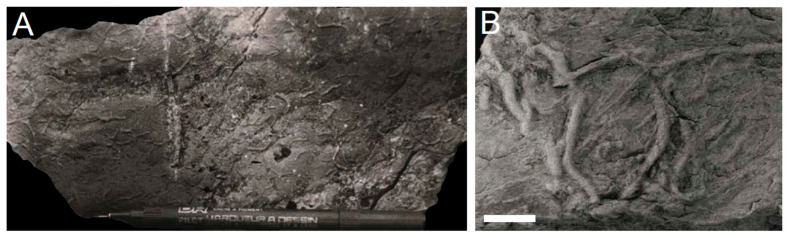
Net-type graphoglyptids from the Ediacaran (**A**) *Multina* and Fortunian (**B**) *Paleodictyon* of Tanafjord. Some of the supposedly most complicated marine trace fossils amidst the earliest record of endobenthic activity. Pen for scale in (**A**) is 1 cm diameter, scale bar in (**B**) 1 cm.

The net-like Graphoglyptida are some of the most complex burrow systems in marine depositional settings. If they were to be created by burrowing, their excavation would require complex “programming” [98] to evolve at or before the Ediacaran–Cambrian boundary since the net-like graphoglyptids are known from the latest Ediacaran (described as *Multina* or *Olenichnus* [3,16,112]; Figure 7).

Modern soft-sediment cores have occasionally recovered shallow-tier polygonal xenophyophore-like protistan organisms [113] comparable to partial *Paleodictyon* and sponge markers have been associated with *Paleodictyon*-like openings [104]. At the same time, however, it is possible for simple organisms such as nematodes and foraminifera to make multi-tiered network burrows comparable to *Multina* isp. [114,115]. Some of the network-like morphology of *Multina* and *Olenichnus* have sharp (unrounded) angles at the branching points of the Graphoglyptida. That lack of corner rounding is common to all *Paleodictyon* and, for this author at least, is very suggestive of branched growth evincing preservation of external molds of an organism rather than being a constantly patrolled burrow. Corner rounding is common in all long trace makers, e.g., worms and some arthropods. Some authors have argued that sharp corners could be maintained in networks if burrowed by a trace-maker that is about as long as the burrow is wide [105], which would need a strange near spherical morphology.

## 6. Conclusions or “What if….?”

The ideas outlined above constitute testable hypotheses that admittedly ask very difficult questions of the rock specimens we have to work with, but should not be discarded in preference for conventional interpretations without careful consideration.

The questions around the demise of the Ediacaran biotas and the diversification of animals in the lower Cambrian are first order paleontological questions. Whether the graphoglyptids function as microbe farms that were patrolled, irrigated and browsed upon by a short-bodied active burrower; or whether they are the external molds of a simple pre-placozoan-grade rangeomorph-like organism that grew in or through the sediment is also key.

If we could know unequivocally what the enigmatic open burrow-like structures in the lowermost Cambrian are, we might become a step closer to understanding either the persistence or otherwise of the chemosymbiotic Rangeomorpha, or better appreciate the paleobiology of the earliest burrows. Either way, it is considered here that they might make a poor choice for delineating the base of the Phanerozoic. The abundant traces of arthropods might be preferable for their lack of ambiguity if nothing else.

The fossil record of the dawn of animal life is full of hints and contradictory evidence, provincialism and incomplete datasets. The questions around the affinities of the Ediacaran biota and the appropriate choice of marker for the Ediacaran boundary are still far from resolved. There is much yet to do, and the hypotheses generated by asking the awkward question ‘What if…?’ are more likely to provide novel answers than not asking.
